# Supplementary material for: A New Cationic Fluorescent Probe for HSO3− Based on Bisulfite Induced Aggregation Self-Assembly
Source: Molecules. 2022 Apr 7;27(8):2378. doi: 10.3390/molecules27082378 (PMC9033099; doi:10.3390/molecules27082378)
Supplement: Supplementary file 1 [file molecules-27-02378-s001.zip › molecules-1657877-supplementary.pdf]

Supporting information for

A new cationic fluorescent probe for HSO_3^- based on bisulfite induced aggregation self-assembly

Xing Zhang[a], Shaoyuan Su[b], Xuan-Ting Chen[a], Ling-Yi Shen[a], Qi-Long Zhang[a]*, Xin-Long Ni[b]*, Hong Xu[a], Zhi-Yong Wang[a]*, Carl Redshaw[c]

[a] School of Public Health, the Key Laboratory of Environmental Pollution Monitoring and Disease Control, Ministry of Education, Guizhou Medical University, Guiyang 550004, China.

[b] Key Laboratory of Macrocyclic and Supramolecular Chemistry of Guizhou Province, Guizhou University, Guiyang 550025, China

[c] Department of Chemistry, University of Hull, Cottingham Road, Hull, Yorkshire, HU6 7RX, U.K.

*Corresponding author: E-mail: sciqlzhang@gzu.edu.cn (Q.L.Zhang) or longni333@163.com (X. L. Ni).

1. Supporting diagram for article

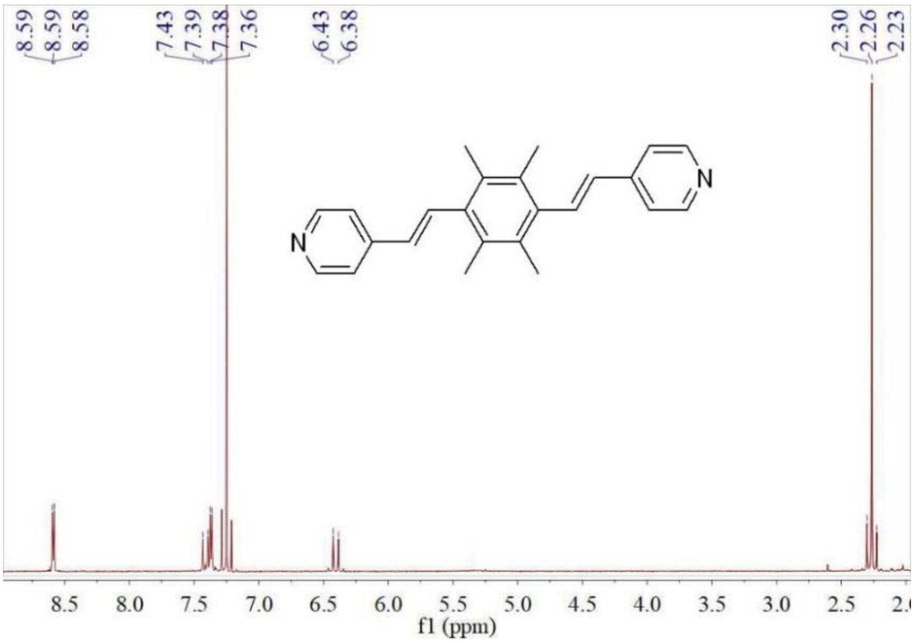

Figure S1. ¹H NMR spectra of TBPD.

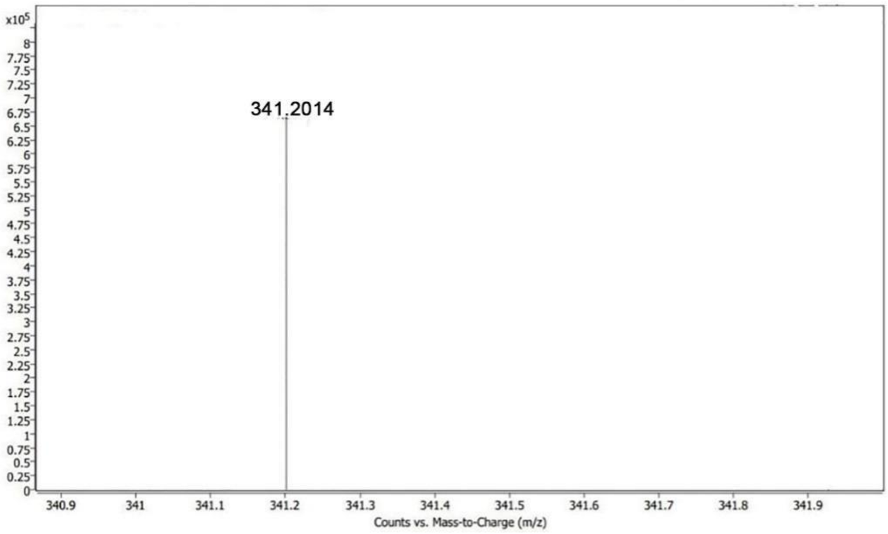

Figure S2. HRMS spectra of TBPD.

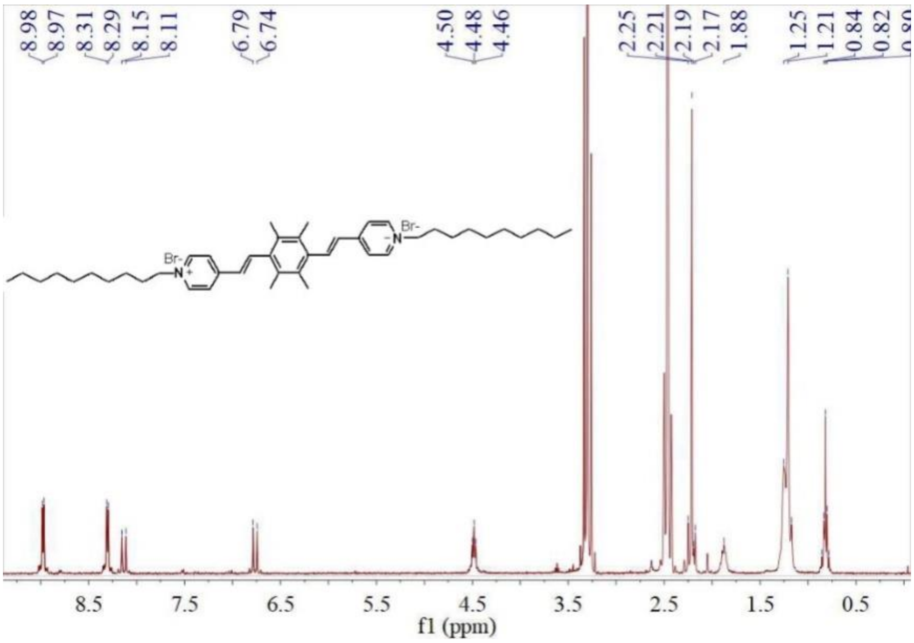

Figure S3. ^{1}H NMR spectra of $TBPD^{2+}$-10C.

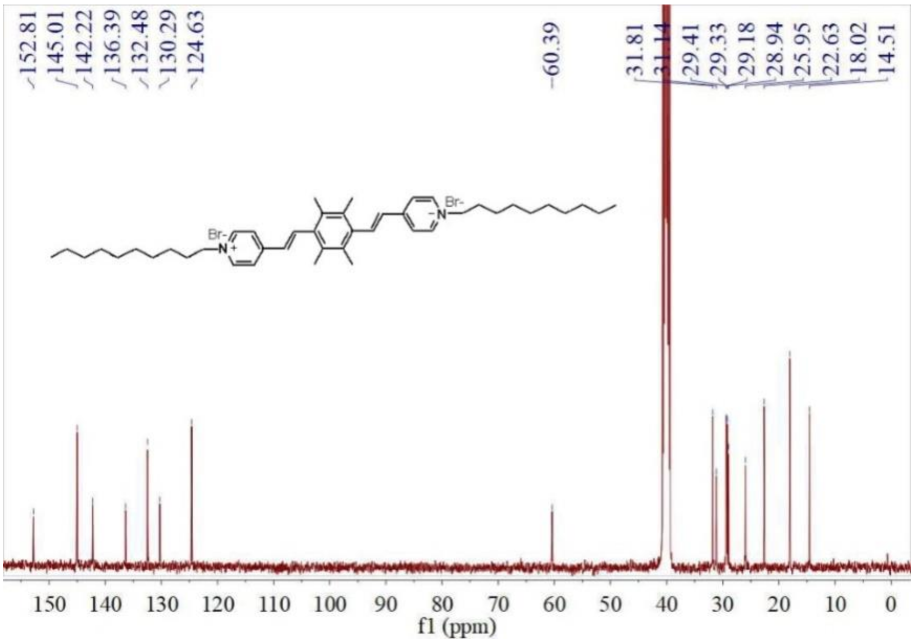

Figure S4. ^{13}C NMR spectra of $TBPD^{2+}$-10C.

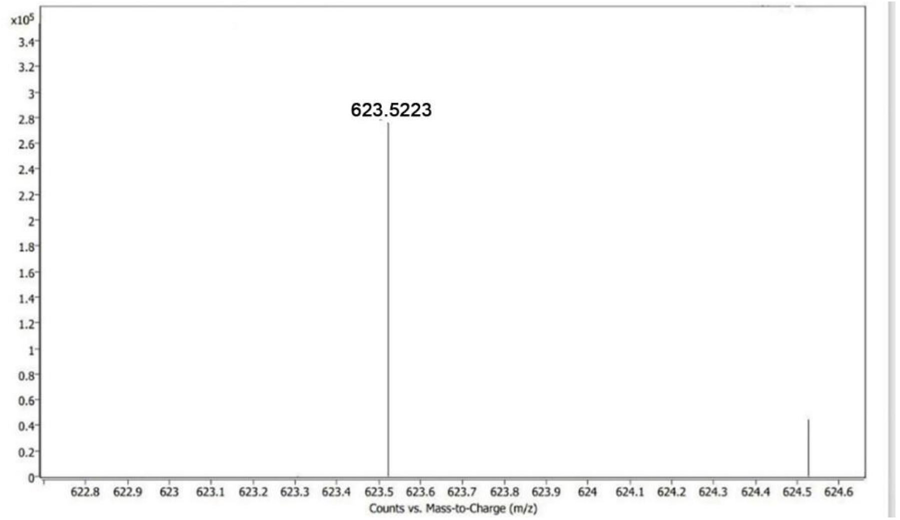

Figure S5. HRMS spectra of TBPD^{2+}-10C.

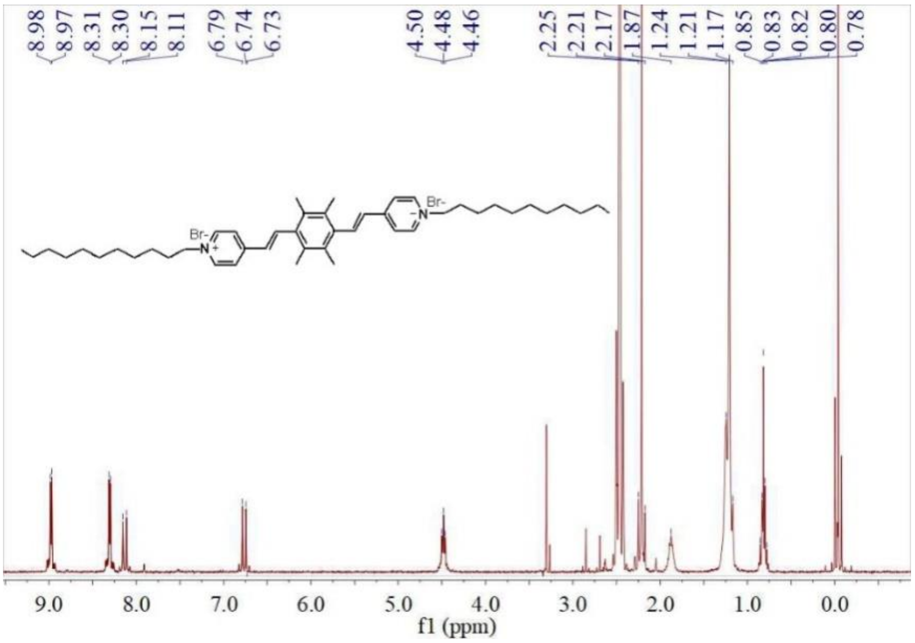

Figure S6. ^{1}H NMR spectra of TBPD^{2+}-11C.

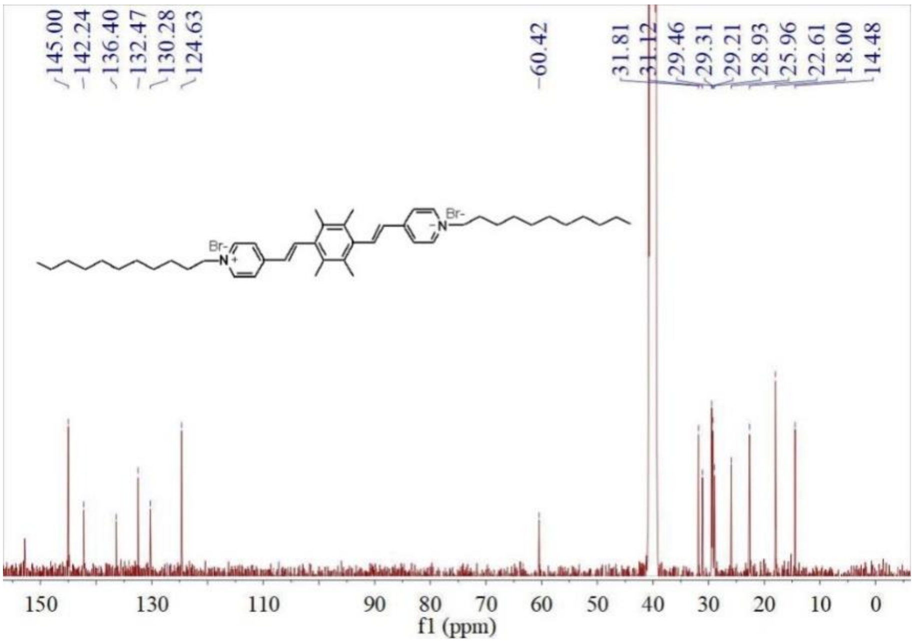

Figure S7. ^{13}C NMR spectra of TBPD^{2+}-11C.

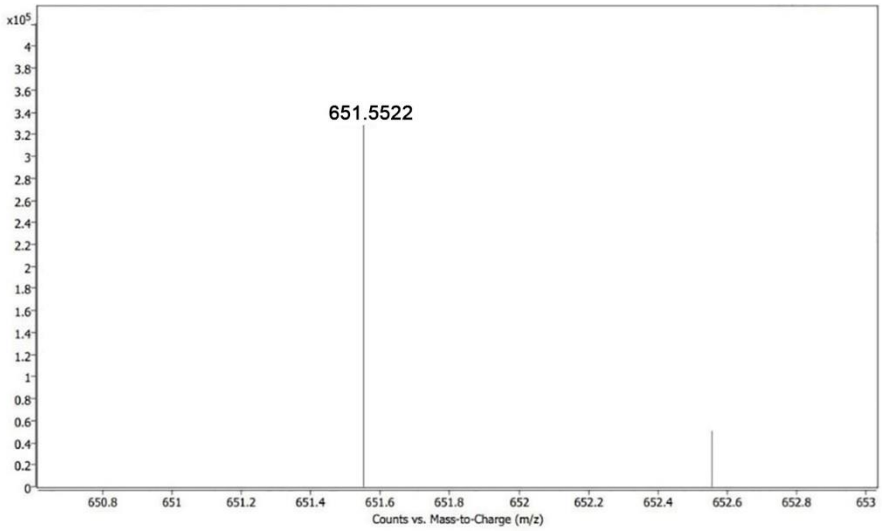

Figure S8. HRMS spectra of TBPD^{2+}-11C.

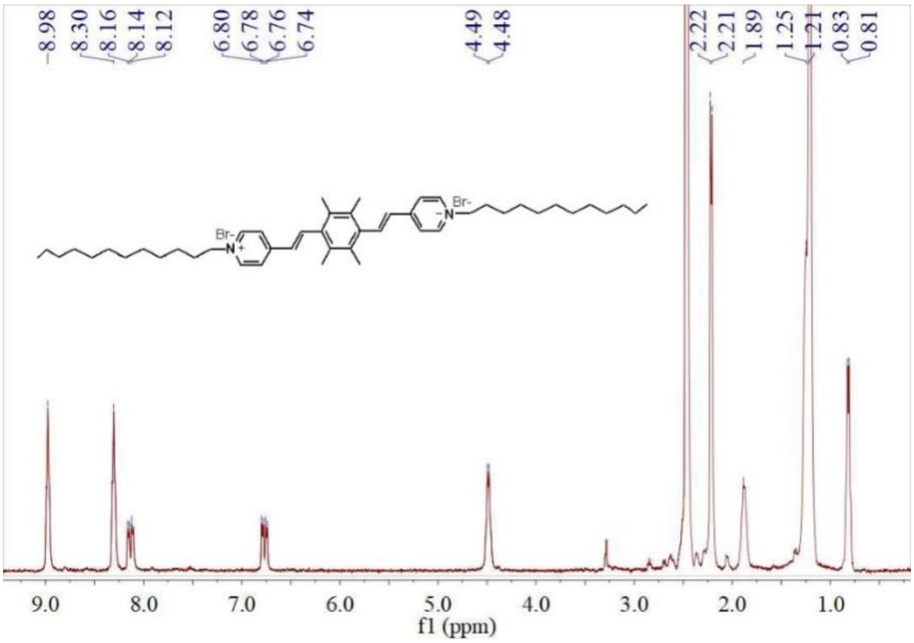

Figure S9. ^{1}H NMR spectra of TBPD^{2+}-12C.

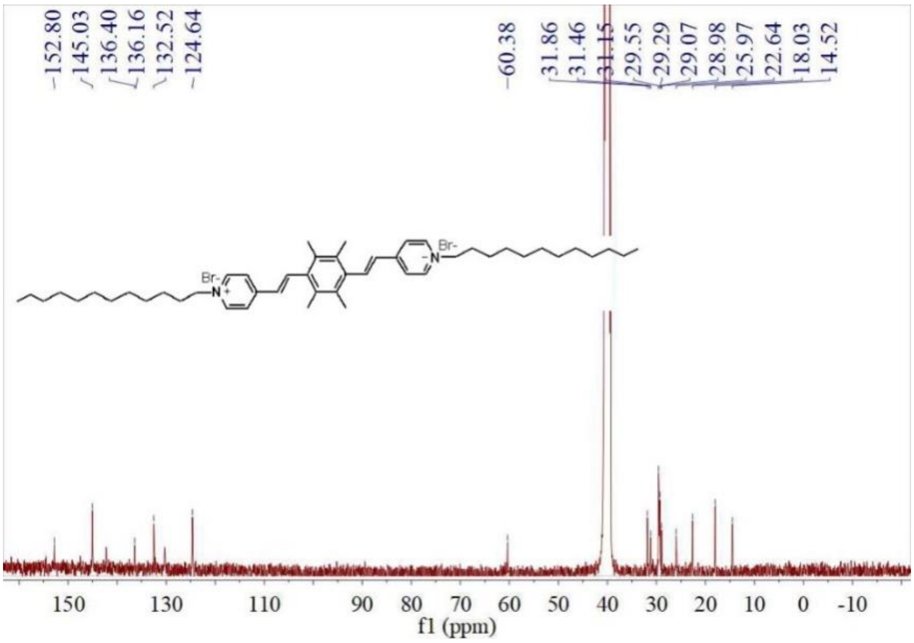

Figure S10. ^{13}C NMR spectra of TBPD^{2+}-12C.

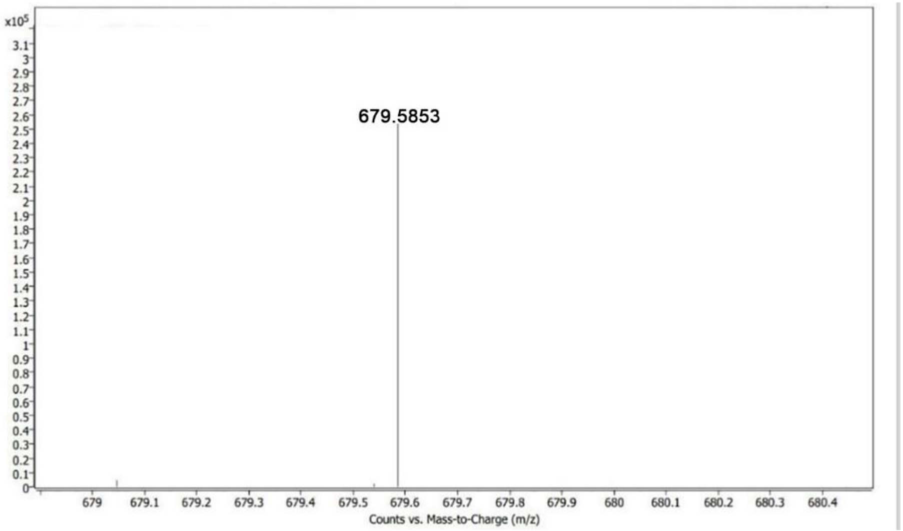

Figure S11. HRMS spectra of TBPD^{2+}-12C.

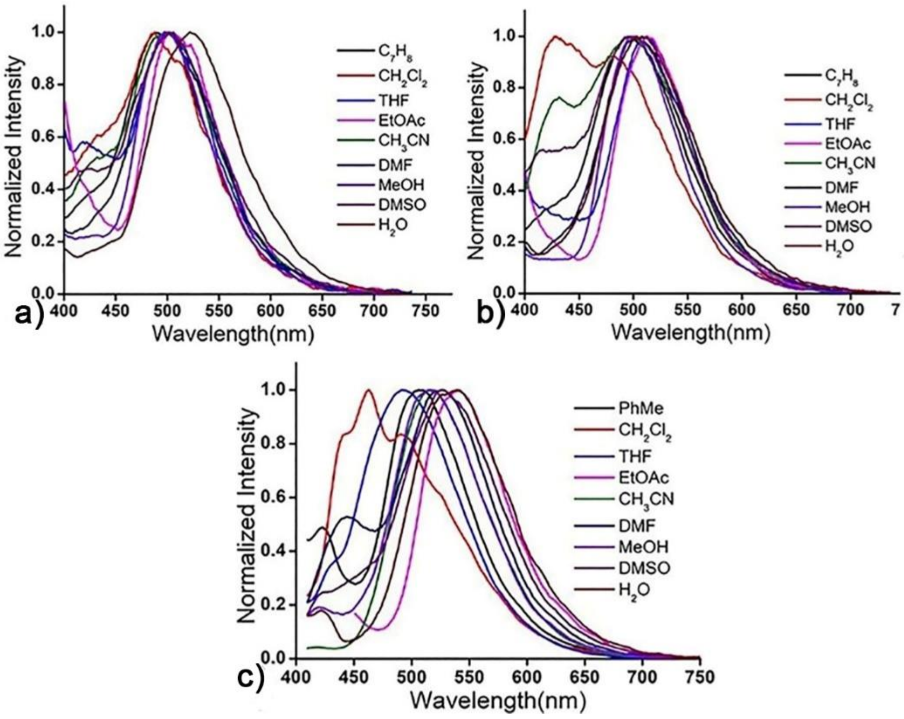

Figure S12. PL spectra of a) TBPD^{2+}-10C b) TBPD^{2+}-11C and c) TBPD^{2+}-12C in different polar solvents (C_{sensor}= 5.0μM, λ_{ex}=348 nm).

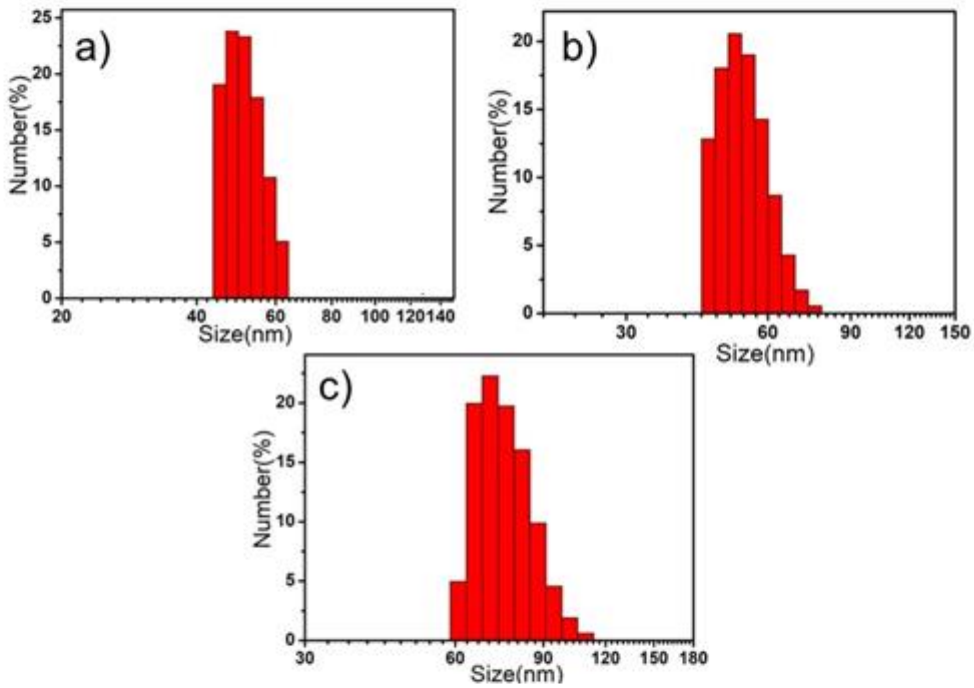

Figure S13. DLS data of a) TBPD^{2+}-10C b) TBPD^{2+}-11C and c) TBPD^{2+}-12C in aqueous condition.

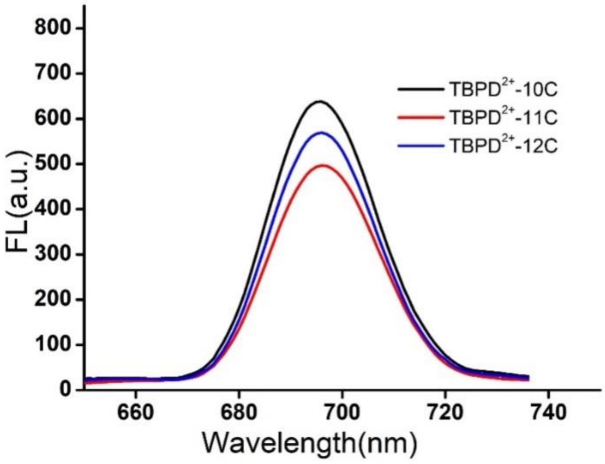

Figure S14. Solid state fluorogram of TBPD^{2+}-10C, TBPD^{2+}-11C and TBPD^{2+}-12C (λ_{ex}=342 nm, voltage: 650V, slit: 5/5 nm, 5.0 μM sensors).

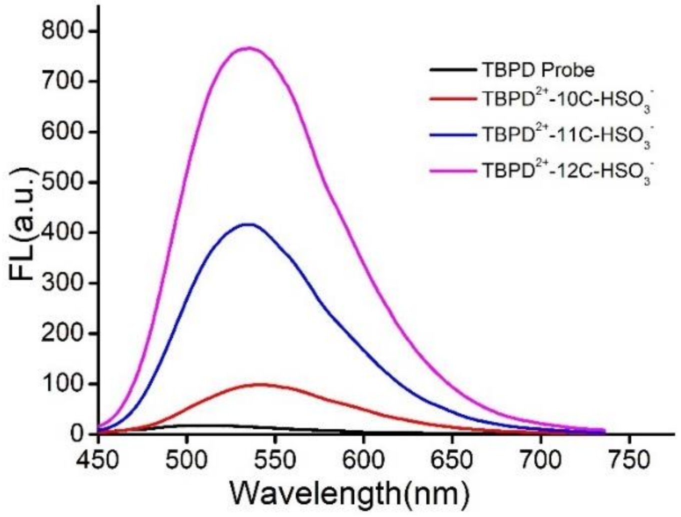

Figure S15. Comparison of the fluorescent intensity for $TBPD^{2+}$-10C, $TBPD^{2+}$-11C and $TBPD^{2+}$-12C after adding HSO_3^- to the probe solvents (λ_{ex}=348 nm, voltage: 620V, 5.0 μM sensors, 100.0 equiv. of HSO_3^-, 2mM Tris-HCl buffer, pH=7.00).

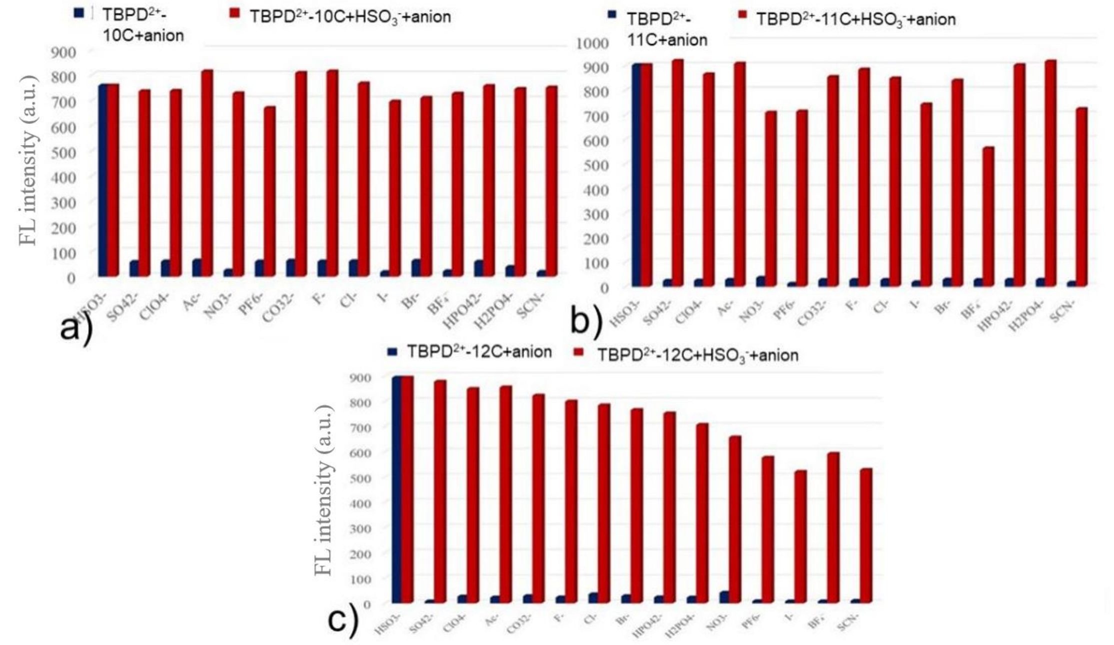

Figure S16. The FL intensity of a) $TBPD^{2+}$-10C b) $TBPD^{2+}$-11C and c) $TBPD^{2+}$-12C to various coexist anions (λ_{ex}=348 nm, λ_{em}=516nm, 5.0 μM sensors, 100.0 equiv. of HSO_3^- and co-exist ions, 2mM Tris-HCl buffer, pH=7.00).

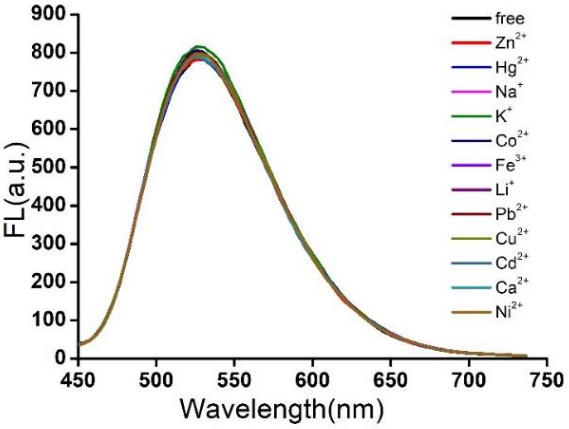

Figure S17. Fluorescent spectra of $TBPD^{2+}$-12C versus different cations (taking $TBPD^{2+}$-12C as an example). (λ_{ex}=348 nm, voltage: 620V, 5.0 μM sensors, 100.0 equiv. of HSO_3^- and co-exist ions, 2mM Tris-HCl buffer, pH=7.00)

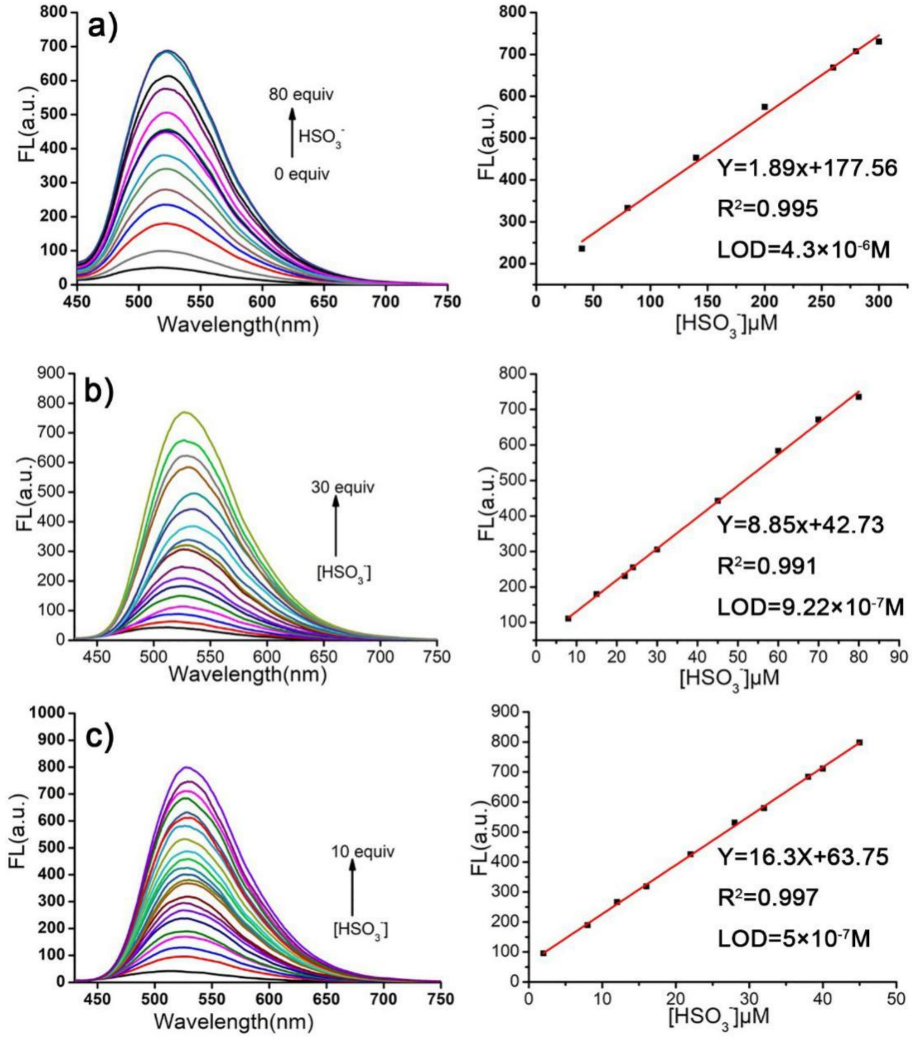

Figure S18. The details of fluorescent titration experiments for a) $TBPD^{2+}$-10C b) $TBPD^{2+}$-11C and c) $TBPD^{2+}$-12C to different concentration of HSO_3^-. (λ_{ex}=348 nm, voltage: 620V, 5.0 μM sensors, 2mM Tris-HCl buffer, pH=7.00)

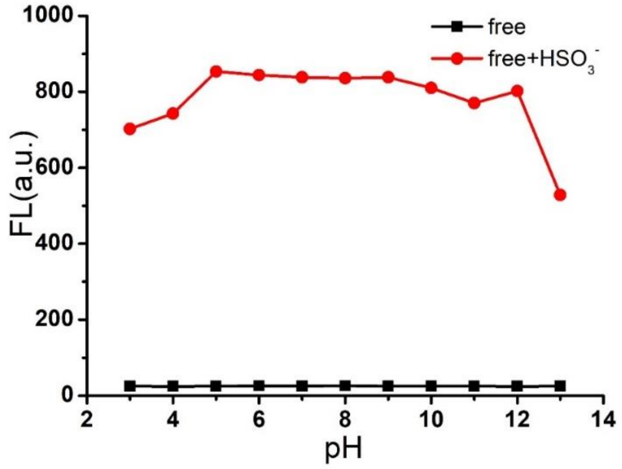

Figure S19. The influence of pH valves on the fluorescent intensity and recognition behavior of TBPD^{2+}-12C and TBPD^{2+}-12C-HSO_3^- system at 537nm (Taking TBPD^{2+}-12C as an instance, λ_{ex}=348 nm, voltage: 620V, 5.0 μM sensors, 100.0 equiv. of HSO_3^-, 2mM Tris-HCl buffer, pH was adjusted by 0.1M HCl and NaOH).

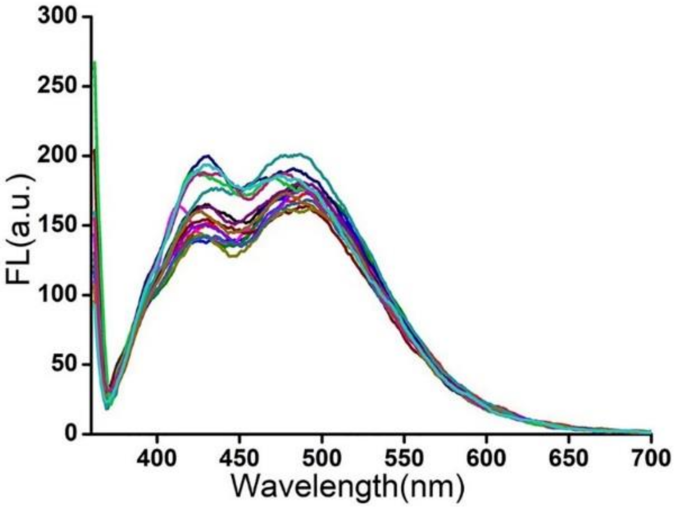

Figure S20. Fluorescent spectra of TBPD^{2+}-12C (5.0μM) towards various anions (such as Cl^-, SO_4^{2-}, SO_3^{2-}, ClO_4^-, Ac^-, NO_3^-, HSO_3^-, PF_6^-, CO_3^{2-}, F^-, I^-, BF_4^-, $H_2PO_4^-$ and SCN^-, 0.5mM) by utilizing MeOH as the recognition medium (taking as TBPD^{2+}-12C an example).
